# Students’ values, professional socialization and the mental gap of corporate social responsibility perceptions

**DOI:** 10.1371/journal.pone.0261653

**Published:** 2021-12-22

**Authors:** Nikša Alfirević, Vojko Potočan, Zlatko Nedelko

**Affiliations:** 1 Faculty of Economics, Business and Tourism, University of Split, Split, Croatia; 2 Faculty of Economics and Business, University of Maribor, Maribor, Slovenia; Universitat Jaume I, SPAIN

## Abstract

This paper examines how values and professional socialization in business schools impact the formulation of students’ contextualized view of social responsibility. We propose the empirical concept of a mental gap between the existing and the wished-for level of a business school’s corporate social responsibility and estimate it empirically by using a sample of business school students from Central and South East Europe. Results show that students wish their business schools to reduce their current orientation toward economic outcomes and focus on environmental and social responsibilities. We interpret those empirical results in terms of the students’ wish to balance achieving economic prosperity and enjoyment of life with the prosocial outcomes of their education. New student generations’ perception of corporate social responsibility is not shaped by the professional socialization patterns but rather by the own perceptions, which can be influenced by experiential approaches to academic teaching and learning. Based on these empirical results, implications for academic practice and future research are explored.

## Introduction

As new generations of students are entering higher education (HE), they bring along their values and views of organizations’ corporate social responsibility (CSR), together with the expectation to learn more about the role of business in society from their HE institution (HEI).

An increasing number of universities and business schools move towards responsible management education, understanding the CSR concept as a trans-disciplinary construct, consisting of the (business) ethics (the economic dimension), responsibility toward stakeholders (the social dimension), and the sustainability domains (the natural, i.e., environmental dimension) [1]. Therefore, it becomes imperative to shed light on students’ values and attitudes on the one hand and on the role that HE plays in shaping them on the other. The role of HE as a professional socialization agent is crucial in developing business leaders [2], who are responsible, ethical, and conscious, thus leading to higher levels of CSR in general. This has been demonstrated by previous studies, analyzing students’ perspectives of business ethics and CSR and estimating the potential business school impact. Even in societies, such as the Finnish one, which could be regarded as highly aware of ethics and proactive in responding to ethical issues [3], business students did not perceive the ethics and CSR to belong to the top of the corporate agenda [4]. Simultaneously, other studies of business students argue that the length of education provides a better attitude toward ethics and CSR [5]. Generalizations still seem to be difficult, as recognized by Waples et al. [6], whose meta-analysis of 25 business ethics programs shows minimal effects compared to the intended outcomes. Similar issues seem to be present across the entire higher education sector, with social responsibility and environmental sustainability education being slowly integrated into mainstream curricula [7], as well as dependent on students’ contingent characteristics [8, 9] and various non-curricular activities and nudges [10].

The (business) ethics and CSR education become even more difficult in the contemporary social context, characterized by the political and ideological divisions and interests [11, 12]. Thus, the theory starts experiencing limitations in framing the moral influence of higher education. The traditional notion of leading students toward a meaningful and competent understanding of complex ethical conflicts [13], by advancing through the pre-defined stages of moral reasoning [14], becomes a somewhat naïve proposition.

However, there is more than black and white thinking. On the one hand, students have already been exposed to a range of contemporary social challenges and ideologies, which have already shaped their moral development. The theory recognizes the limit to opportunities for changing ethical attitudes in higher education, although this position received mixed empirical support [15, 16]. On the other hand, the context of moral imagination and a range of other individual factors also count regarding students’ future ethical behavior [17], which can be influenced by using narrative teaching methods and reframing students’ personal experiences [18].

This study addresses the gap in the extant literature, which finds it difficult to comprehensively address the ethical views held by new student generations, i.e., Generation Y (‘Gen Y’, or the ‘Millenials’), born in the 1980–1995 period and the Generation Z (‘Gen Z’, or the ‘Zoomers’), born in the 1995–2015 period [19]. Some empirical findings have described the individual aspects of these generations’ moral attitudes and behaviors. For instance, the ‘Gen Y’ members value actions more than proclamations, as CSR activities directly influence their CSR-related behavior. For older generations, there is only an indirect relationship between CSR perceptions and the behavioral response [20]. This finding shows that starting with the ‘Gen Y’, a clear path of moral development, as described by Kohlberg and Hersh [14], might not be applicable anymore. This view is supported by findings, which show different ethical evaluations within the ‘Gen Z’. They are described as both inclusive and authentic [21, 22], constructive and oriented toward the work-life balance [23], but also as increasingly pessimistic, with a lower civic orientation and distrust of social institutions [24, 25]. These extant literature contradictions could lead to the proposition that personal experiences and other factors become much more critical in shaping their ethical views. We believe that researchers should acknowledge the importance of unique student characteristics and develop the guidelines for academic teaching and learning in the fields of ethics and CSR, based on the idea that moral education has the potential to reach its objectives [26, 27].

Suppose personal experience plays a crucial role in influencing CSR attitudes and shaping future CSR-related behavior. In that case, there is a need to consider both the individual students’ characteristics, such as values, with the educational approaches and professional socialization.

The effects of education received in business schools (i.e., responsible management education–RME), framed by using the notion of professional socialization, which might be secondary to the gendered stereotypes [4], but still influences students’ attitudes and behaviors. Haski-Leventhal, Pournader, and McKinnon [9] show a direct relationship between RME and students’ CSR intentions and a serial mediation mechanism consisting of values and CSR attitudes.

However, it is not clear if the currently identified patterns of professional socialization still work for the new student generations. This can be done by addressing the following research questions:

- How do new generations of business school students assess CSR when using their school as the organizational benchmark?
- What is the influence of individual characteristics versus the impact of professional socialization when students assess their schools’ CSR?

These research questions are explored by using Schwartz’s values theory and estimating professional socialization effects in business schools on the sample of 532 students from Central and South East Europe (CEE and SEE). The study aims to explore the students’ mental gap between the personal assessment of the required and the current CSR level of their school. In doing so, the paper contributes to explaining the role and sources of personal experience, as used by new generations, in their assessment of organizational CSR.

We believe that this study contributes to the extant literature by pointing to the line of research, acknowledging the factors that drive the individualization of students’ ethics and CSR while allowing for the influences of the educational environment and professional socialization. We emphasize the need to let the students discover and negotiate CSR-related attitudes and behaviors as one of the most important implications for academic practice.

This could prove to be especially valuable in the CEE and SEE social contexts and environments since those seem to be plagued with public distrust of official institutions, often replaced by reliance on personal networks, friends, and family [28]. In such an environment, public distrust seems to represent a historical and institutional continuity [29]. Therefore, official proclamations of (business) ethics and CSR, by delegitimized social and educational institutions, will probably be met with cynicism and disillusionment [30] unless young people are provided with the opportunity to co-create the notion of what CSR should be. In addition, business schools and other HEIs will be used as CSR benchmarks in the formulation of CSR attitudes and behavior of young people in their workplaces, and the obtained empirical results prove to be valuable for the study of the future patterns of CSR in the corporate environment, as well.

## Students’ values and business school socialization

The classical ‘nature vs. nurture’ debate on sources of individual ethics is a popular approach, differentiating between personal effects and those derived from the institutional context, with solid empirical support in the fields of economics and business [31]. This presupposes that students’ fundamental values will influence the evaluations of CSR actions since they represent principles guiding individual behavior in various situations [32]. Personal values are a non-prescriptive framework, allowing for different interpretations. However, they can be linked to prescriptive ethics models, such as Kohlberg’s moral reasoning stages [33] and personal moral philosophy [34]. The additional benefits of personal values, as predictors of individual attitudes and behavior, include stability during young adulthood [35] and across cultures [36].

The body of research on the influence of personal characteristics on students’ CSR attitudes recognizes that specific value profiles could lead to a higher level of CSR orientation. However, there does not seem to be an identified value pattern. Fukukawa et al. [37] empirically identified two factors of social and environmental responsibility (enterprise CEOs’ commitment and government regulation) and associated them to MBA students’ values of universalism. Bhattacharyya [38] has confirmed their findings and found additional CSR influence on the security and benevolence of personal values. Ng and Burke [39] reported a higher level of CSR orientation for US business students, showing a higher level of Rokeach’s social value. Wang and Juslin [40] linked altruism-related values to Chinese students’ perceptions of CSR performance. An analysis of a business undergraduate sample at a Brazilian university led to a profile of self-direction, stimulation, universalism, and benevolence values associated with a higher level of CSR orientation [41]. The diverse and inconsistent patterns of empirical findings found by these studies regarding the relationship between personal values and CSR orientation(s) suggest a range of different but potentially generalizable patterns, dependent on the students’ characteristics and their social circumstances.

The social circumstances include the influence of students’ socialization within the socio-cultural context of the business schools, which has been extensively discussed in the academic literature [2, 4, 43–45]. Educational institutions influence the development of different social competencies, including ethical ones. They provide opportunities for social interactions and boundaries of what is considered socially and culturally appropriate, leading to balancing positive outcomes for individual students within the institutional expectations of acceptable behavior [42]. Therefore, it is possible to study different levels and types of educational institutions’ influence on students’ values, attitudes, and behaviors.

In recent literature, organizational socialization within a business school, as a source of cross-generational transfer of professional practices [43], including ethics and CSR, has been studied by Lämsä et al. [4]. They suggest that the business schools (at least in the Nordic socio-cultural context) seem to promote the stakeholder concept into the mainstream. However, they might not be doing the same with the idea of equal opportunity employment. When comparing the influence of studying business vs. studying social work to student values, Arieli, Sagiv, and Cohen Shalem [2] found that the self-selection is based on a value fit between the individuals and their future professions. In contrast, the influence of professional socialization on personal values is somewhat limited. However, Haski-Leventhal, Pournader, and Leigh [44] have noticed that these results imply driving business students away from the prosocial values of traditional education. Rosengart, Hirsch, and Nitzl [45] confirm the students’ self-selection into their academic studies based on personal values and show that professional socialization, as a contextual factor, influences students’ decision-making as they progress through their academic programs.

The potential generalization of these studies [2, 43–45] seems to hint at a limited influence of business schools on students, who tend to self-select, based on the perceived compatibility with the value profile of the profession and, eventually, the attended school. However, it is still open for discussion, if there is, and how significant the potential to change business students’ values is. In addition, the generalizability of the factors related to the business school as a socialization environment should be highly sensitive to the educational systems and cultural circumstances, which limits empirical research to students and business schools from similar educational and cultural contexts.

There is a limited number of studies touching on the process and outcomes of (under)graduate students’ professional socialization, with the work of Weidman [46] and its conceptual development [47] being considered the focal points of the extant literature. The generalized Weidman’s model acknowledges multiple socialization agents and processes, with the business school’s influence recognized in normative contexts, created by the discipline (department), curriculum and peer groups, and socialization processes (learning, social interactions, and integration).

Although we are not aware of previous empirical work on the socialization of business school students in CEE and SEE contexts, a study on the interactions between the strategic orientation of business schools and their CSR is available [48]. It conceptualizes the socialization influences to students in terms of quality of education, fostering the culture of students and HE organizations, social goals, and flexibility of education. These constructs are based on the values of the higher education system, oriented toward the creation of knowledge and its alignment with the needs of the society, as well as the social orientation of students and HE institutions. They could be compared to Weidman’s model regarding socialization goals.

As indicated in the introduction, both socialization goals and the process could be impaired by social distrust and cynicism, implied by the CEE and SEE social contexts. Suppose official proclamations of CSR are ignored or even met with a resistance, based on the negative assessment of post-communist history and public policies [49]. In that case, there will be a failed socialization of young people–both into their professional and social roles. This context results in grave socio-economic consequences, including the mass migration of young people and the lack of reconciliation in post-conflict societies, such as Bosnia and Herzegovina. The contextualization of research results will be provided in the discussion section.

## Predictors of CSR: Research proposition and hypotheses development

The notion of a gap between the idealized view of ethics, held and preached by HEIs, and job realities is nothing new in business education and practice [50, 51], along with assessing how ethics courses might influence an ethical gap [52]. The positivistic research tradition believes that it is possible to generalize the factors causing or influencing such a gap within the student body [53, 54] or tools/teaching methods, which could be helpful to eliminate it [55]. However, there seem to be severe problems with contemporary business schools, slowly introducing change and still emphasizing the classical notion of the rational, profit-making business paradigm instead of considering how to improve the human conditions in challenging times [56].

This disparity leads to whether it makes sense to teach and research (business) ethics and CSR. In case of a positive answer, one might resort to the postmodern concept of rejecting the ethics and CSR as ‘grand narratives’, based on the interests of the privileged social groups to suppress local stories and alternative views [57], coming from the otherwise suppressed and unrepresented actors. This would entail the fluid and individual subjective interpretations of the organizational CSR [58] and, thus, jeopardize the pragmatic truth-seeking orientation of social theory [59].

Instead, this study opts for a contextualized approach, examining both the individual and socialization influences on new business student generations’ CSR attitudes and behaviors. Answering the call of Härtel and O’Connor [60] to incorporate the relevant but previously unexplored context(s) into organizational research, as to create new insights, we acknowledge both the personal and the social context of the study.

In addition, we consider the specific socio-economic environment in which the research is conducted, which can influence both the individual and the social dimensions of the research context. In this study, the CEE and SEE countries’ socio-economic environment (Slovenia, Croatia and Bosnia and Herzegovina) seems to be quite varied, although all based on the shared cultural and institutional patterns.

The three countries belonged to the two Yugoslav states, throughout most of the 20^th^ century (1918–1941 and 1945-1991/1992), with the last three decades being characterized by the development of national states, markedly different in their success of overcoming the legacies of socialism and the regional conflicts of the 1990s, implementing democratic and economic transition, as well as the EU accession. The EU influence on the SEE region has been uneven, unlike in Central and Eastern Europe, as the local conditions and national priorities mitigated the European initiatives [61]. Since a comprehensive analysis of the socio-economic circumstances, including the relevant human and sustainable development characteristics, in the CEE and SEE should be conducted separately, in this paper, we provide a limited presentation of the fundamental social pertinent indicators. Those have been drawn from the extant literature related to world values [62], human [63], and sustainable [64] development (see Table 1). Additional contextualization of research results will be provided in the discussion section of this study.

**Table 1:** Selected socio-economic indicators, including human and sustainable development of Slovenia, Croatia, and Bosnia and Herzegovina.

Selected indicators of socio-economic, human, sustainable development	Slovenia	Croatia	Bosnia and Herzegovina
Human development index (HDI) and its components *(Human Development Report 2020*)			
HDI value 2019	0.917	0.851	0.780
HDI rank 2019	22	43	73
Change in HDI rank 2014–2019	+2	+2	+8
Average annual HDI growth (1990–2019)	0.59	0.79	N/A*
Life expectancy (years)	81.3	78.5	77.4
Gross national income (in 2017 PPP USD)	38,080	28,070	14,872
**Selected values indicators *(World Value Survey Wave 7*, *2017–2020)***			
Feeling ’very happy’	21%	18.5%	29.6%
A feeling of a ’great deal’ of choice and control in life	19.9%	22.5%	34.4%
Being ’completely satisfied’ with one’s life	14.3%	21.9%	29.7%
Public trust: Most people can be trusted	25.3%	13.6%	9.6%
’A great deal of confidence’ into government	1.7%	0.8%	3.1%
’A great deal of confidence’ into major companies	4.5%	2.8%	6.7%
’A great deal of confidence into the environmental protection movement	6.2%	5.2%	7.7%
Absolute preference of income equality over income differences	14.9%	24.5%	32.1%
Absolute preference of government ownership of the businesses	5.9%	19.9%	35.2%
Protection of environment preferred to economic growth	60.2%	52.8%	36.7%
Absolute preference of government taking responsibility for social welfare	8.7%	18.8%	27.2%
A feeling of the country being governed ’not at all democratic.’	6.5%	17.2%	30%
Feeling ’very proud’ about their nation	50.8%	39.2%	28.4%
**Sustainable development index in Sout East Europe—*as calculated by Golusin and Munitlak Ivanović [64]***			
Overall value	611,638	396,531	224,271
Economic dimension	538,000	310,320	130,350
Environmental dimension	63,100	80,040	90,070
Social dimension	10,104	5,645	3,535

* The average annual HDI growth for the 2000–2010 period equals 0.66, and for 2010–2019 equals 0.88.

We address the previously discussed aspects of the research context by incorporating a mental gap variable into our empirical strategy (see the next section of this study for a complete discussion), according to Griffin’s [65] ‘second tier’ contextualization strategy.

In addition, the concept of a ‘mental gap’, discerning ‘what is’ from ‘what should be’ in the organizational CSR, is compatible with the affirmative postmodern research [66]. When related to organizations to which respondents are well acquainted (such as their HEIs), this type of research can avoid ‘too much’ generalization and pragmatically describe different levels or groups of respondents’ subjective CSR interpretations. Simultaneously, it places the empirical results within the existing body of knowledge, which shows CSR patterns, depending on students’ personal and other contextual predictors [9, 67]. Therefore, we propose that the mental gap construct should be used to describe the CSR of business schools and how their students perceive it:

H1. There is a significant mental gap in students’ perceptions about the existing and desired state of their business schools’ CSR, showing that business schools are not pursuing their social and environmental responsibilities.

We also predict that the existence of a mental gap between ‘what is’ and ‘what should be’, as related to business schools’ CSR, could be lowered or eliminated by allowing students to make their assessment of CSR and how it should be implemented in educational and business practice. This could be done by a range of different teaching and learning interventions, which are not within the scope of this study. These include service-learning [68], storytelling [69], and other forms of experiential teaching and learning, allowing students to seek their assessments and interpretations of the CSR concept [70]. They are well covered by the extant literature and can be integrated into holistic teaching and learning frameworks [71].

As pertaining from Hypothesis 1, when the economic (financial), social, and environmental sustainability dimensions of CSR are considered [1, 72]:

H1.1. There should be less emphasis on the economic success of business schools.
H1.2. The environmental and social responsibilities of business schools should be less based on the attempts to socialize students into the CSR concept but rather to seek authenticity, based on students’ values and perceived concerns.

## Methods

### Sample and data collection

The sample for this study consists of 524 responses from undergraduate business students in CEE, based on business students from Slovenia (N = 180), Croatia (N = 153), and Bosnia and Herzegovina (N = 191). Respondents in each country were selected using convenience sampling. The survey was conducted in 2019/2020 and included business students from diverse years and specializations. Three different countries from CEE and SEE regions were selected for the empirical research to reach the representative ‘mix’ of national and cultural contexts. Empirical work in a single country could not be considered representative because of the diversity of institutional and contingency factors influencing the regional developments during the transition and post-transition periods (i.e., in the last 30 years).

In Slovenia, research was conducted at the University of Maribor, with most responses from students at the Faculty of Economics and Business. In Croatia, most respondents studied at the University of Split, Faculty of Economics, Business, and Tourism. At the same time, most responding students from Bosnia and Herzegovina are enrolled at the University of Banja Luka, Faculty of Economics. In Slovenia, surveying was conducted during classes with a paper-based questionnaire, and students participated voluntarily.

It should be noted that most Slovenian respondents are bachelor-level students. On the one hand, we acknowledge a limitation of the Slovenian sub-sample, consisting primarily of bachelor students, who might not be well informed of the challenges of CSR. On the other hand, since our research seeks to understand the CSR perceptions of new generations, this feature of the Slovenian sub-sample somewhat corrected the potential age bias, coming from the Croatian and B&H sub-samples, with a considerable number of master students enrolled into their fifth year of study. Our teaching and administrative experience show that some of those students could be postponing their graduation to keep some of the students’ social benefits until a desirable job is found.

The first page of the questionnaire informed the participants about the purpose of data collection, assured their anonymity and the usage of data for scientific purposes only, and asked for consent to participate in the survey. In Croatia and Bosnia and Herzegovina, surveying was conducted using a Web-based tool. The first Web page contained the same information and the consent requirements as used in the Slovenian paper-based questionnaire. No personally identifiable information was collected in both cases, including names, student IDs, or IP addresses. Detailed sample characteristics are outlined in Table 2.

**Table 2:** Demographic characteristics of the respondents.

Characteristic	Slovenia	Croatia	Bosnia and Herzegovina
	(N = 180)	(N = 153)	(N = 191)
Gender			
Male	34.6%	24.8%	27.2%
Female	65.4%	75.2%	72.8%
Level of study			
Bachelor	99.4%	61.3%	45.5%
Master	0.6%	38.7%	52.4%

### Constructs and measures

The fundamental theoretical construct in this study is the contextualized assessment of own business school’s CSR, measured by using the comparison of assessments of the current CSR level (referred to as the ‘IS’ level) vs. the individual perception of the ideal organizational CSR level (referred to as the ‘SHOULD’ level), concentrating on the specific attitudes toward organizational CSR actions, instead of assessing the general statements about the CSR. Dimensions for CSR assessment were adopted from Furrer et al. [73] and Ralston et al. [74]. We used this specific conceptualization due to its previous partial empirical verification in the cultural environments of CEE and its history of usage in the HE studies across the CEE region(s). Respondents were first asked to assess the current state of their business school (the ‘IS’ state), while, in the following set of questions, they were asked to evaluate the ideal form of their school’s CSR (the ‘SHOULD’ state). The CSR perceptions were structured into three dimensions (economic/financial, environmental/natural, and social), as outlined in the theoretical part of the study, and measured on a 5-point Likert scale, ranging from 1 (strongly disagree) to 5 (strongly agree).

The measurements were verified by a confirmatory factorial analysis for the current (‘IS’) state (KMO = .964, Bartlett’s test of sphericity = 5030.40; df = 300; p < 0.001), and the desired (‘SHOULD’) state of CSR in HE (KMO = .886, Bartlett’s test of sphericity = 3849.97; df = 300; p < 0.001), we formed variables, reflecting three dimensions of CSR. Details about factor loadings, average variance extracted (AVE), composite reliability (CR), and Cronbach’s alpha coefficients for three variables indicating current state and three indicating desired state of CSR in HE are outlined in Table 3.

**Table 3:** Latent variables, measurement items, factors loadings, AVE, CR, and Cronbach’s alpha for CSR of business schools.

	Current state—IS				Desired state–SHOULD			
	Factor loadings	AVE	CR	Cronbach’s α	Factor loadings	AVE	CR	Cronbach’s α
**Financial (economic) responsibility**		.633	.775	.581		.523	.687	.603
1. Worry first and foremost about maximizing profits	.770				.733			
2. Always be concerned first about economic performance	.821				.713			
**Environmental responsibility**		.606	.687	.853		.560	.792	.795
1. Prevent environmental degradation caused by the pollution and depletion of natural resources	.755				.763			
2. Adopt formal programs to minimize the harmful impact of organizational activities on the environment	.829				.769			
3. Minimize the environmental impact of all organizational activities	.749				.710			
**Social responsibility**		.418	.740	.795		.291	.619	.724
1. Contribute actively to the welfare of our community	.711				.537			
2. Plan for their long-term success.	.650				.484			
3. Help solve social problems	.555				.620			
4. Train their employees to act within the standards defined by the law	.659				.506			

Values, as individual predictors of CSR in HE, were conceptualized and measured by using the short version of the Schwartz Value Survey (SVS) model [32], as formulated by Ralston et al. [75] and rated by using a 9-point Likert-type scale, ranging from “of lowest importance for me” (1) to “of supreme importance for me” (9). The ten individual sub-dimensions of personal values proved to have a high level of internal consistency, measured by the Cronbach alpha: power (α = .643), achievement (α = .664), hedonism (α = .567), stimulation (α = .704), self-direction (α = .665), universalism (α = .765), benevolence (α = .768), tradition (α = .614), conformity (α = .670) and security (α = .665). The measures used have been confirmed as robust across individuals and national cultures [76] and previously validated in studies of the HE sector [38, 40, 41].

Socialization influence of businesses schools, considered relevant for convergence of individualized CSR perceptions were previously considered by Popović and Nedelko [48]. As previously discussed, the three CEE and SEE countries in which the empirical research has been conducted are comparable from the cultural, historical, and institutional viewpoints, but diverse enough to represent various contextual circumstances across the region.

We adopted the items from their study, measured on a 5-point Likert scale, ranging from 1 (strongly disagree) to 5 (strongly agree). Nevertheless, following theoretical argumentations from the third section of this study, we strived to identify a smaller number of determinants, which would be useful in terms of a frame for patterning the behavior of individual students (and other actors in the future research). Based on the exploratory factorial analysis (see Table 4), we formed two new constructs, labeled ‘Development of knowledge and competencies’ and ‘Culture development’, aiming to describe the results of the business school socialization process in terms of relevant business knowledge and skills developed, versus the prosocial outcomes achieved. Statistical results show that the new variables are formally acceptable (KMO = .956, Bartlett’s test of sphericity = 5300.62; df = 153; p < 0.001). Details about factor loadings, AVE, CR, and Cronbach’s alpha coefficients for two variables measuring business school socialization are outlined in Table 4.

**Table 4:** Latent variables, measurement items, factors loadings, average variance extracted (AVE), composite reliability (CR), and Cronbach’s alpha for business school socialization.

	Factor loadings	AVE	CR	Cronbach’s α
**Development of knowledge and competencies**		.460	.836	.888
1. Excellent knowledge base, emphasizing »knowledge society« and life-long learning	.676			
2. Development of solidarity, as related to the family, minorities, poor, older people, natural environment, and the entire society.	.667			
3. Building an individual’s personal, cultural and national identity (e.g., respecting diversity, national heritage, culture, etc.)	.662			
4. Responsibility through active participation of students in social life and society	.760			
5. High quality of education, by ensuring prerequisites for achieving highest educational standards	.716			
6. The scientific validity of education corresponding with the needs of the modern economy and society.	.576			
**Developing culture of business schools**		.354	.813	.893
1. Equal opportunities for business education by respecting criteria, which will enable equal opportunities for diverse ethical, gender, and national groups	.520			
2. Inclusion of everyone in the education system by respecting educational needs of all stakeholders of education, especially those with special needs and marginal groups	.530			
3. Respect for the human rights and dignity of every person	.639			
4. Professional ethics by ensuring the professionalism of education professionals and their responsivity to work and community	.670			
5. Democracy, by the involvement of key actors in the development of education	.620			
6. Promoting an innovative organizational climate in business schools	.620			
7. European dimensions of education	.584			
**8.** Interculturality, achieved by encouraging understanding and acceptance of cultural differences	.561			

KMO values in CFA and EFA above 0.8 indicate that the factorial analysis is appropriate for understanding examined variables’ structure [77].

### Instrument validation

In terms of the internal validity, the Cronbach’s alpha values for latent variables, related to environmental/natural CSR dimension (IS and SHOULD), social CSR dimension (IS and SHOULD), development of knowledge and competencies, as well as the development of ‘culture’ (as related to the students’ prosocial attitudes), were above the suggested cut-off point of 0.7 [78]. The economic/financial CSR dimension (IS and SHOULD) values were just below or achieved the minimum cut-off point of 0.6, respectively [79] and were comparable to those in studies using the same scale for measuring this variable. For instance, Furrer et al. [73] reported reliabilities for the economic aspect of CSR between .41 and .69 across different countries. In contrast, Potočan, Mulej, and Nedelko [79] reported a value of .61 for employees’ attitudes toward the economic environment. Reliabilities were comparable to studies using SVS dimensions of values in terms of personal values. For instance, Ralston et al. [75] reported similar mean Cronbach’s alpha for 50 societies, similar to our study, i.e. power (α = .65), achievement (α = .66), hedonism (α = .52), stimulation (α = .65), self-direction (α = .61), universalism (α = .77), benevolence (α = .70), tradition (α = .58), conformity (α = .66) and security (α = .60).

Factor loadings were ranged between .520 and .829 and are above the cut-off value of 0.40, reported in a paper comparing 37 studies published in four psychological journals [80]. Regarding convergent validity of measures, the CR values were above .619, thus, higher than the commonly used threshold of .600 [81]. The AVE ranged from .291 to .633, which is below the recommended level of .500, although AVE is a more conservative measure of the validity of measures [81]. Thus, a researcher could use the CR value alone to conclude that the convergent validity of the variable is adequate, although more than 50% of the variance is due to an error. For instance, Lam [82] reported AVE above .310 as acceptable. As CR values are above thresholds and AVE are comparable to other surveys, we can conclude that our measures are reliable.

### Common method variance measures

Since we had sources of both the independent and dependent variables in one instrument, the possibility of bias exists [83]. We estimated the common method variance utilizing exploratory factor analysis in SPSS by loading all the items, describing the current and desired CSR state and the two outcomes of the business school socialization (see Table 4), onto a single factor. They were constrained so that there was no rotation [83]. The newly introduced common latent factor explained 24.581% of the variance, which is way below a threshold value of 50% [83]. Also, the correlations among variables of interest in this study (Table 5) were all well below extremely high (>.90), which indicated a slight possibility of common method bias [78]. We can sum up that the possibility of common method bias in this study is not an issue.

**Table 5:** Mean values, standard deviations and correlations among the study variables^a^.

Variable	M	SD	1	2	3	4	5	6	7	8	9	10	11	12	13	14	15	16	17
**Personal values**																			
1. Power	5.315	1.403	1																
2. Achievement	6.975	1.100	.517***																
3. Hedonism	7.225	1.460	.327***	.359***															
4. Stimulation	6.469	1.402	.306***	.463***	.397***														
5. Self-direction	7.387	.991	.242***	.616***	.363***	.547***													
6. Universalism	6.871	1.103	.082	.391***	.198***	.458***	.626***												
7. Benevolence	7.411	1.115	.045	.409***	.258***	.320***	.557***	.580***											
8. Conformity	7.191	1.199	.243***	.411***	.223***	.222***	.419***	.480***	.631***										
9. Tradition	5.893	1.338	.211***	.318***	.036	.177***	.361***	.459***	.515***	.582***									
10. Security	6.981	1.199	.281***	.352***	.317***	.293***	.389***	.469***	.475***	.575***	.443***								
**Business school socialization**																			
11. Development of knowledge	3.319	.895	.181***	.186***	.243***	.150**	.159***	.142**	.159***	.207***	.094*	.238***							
12. Culture development	3.445	.809	.162**	.147**	.251***	.148**	.163***	.152**	.196***	.207***	.088*	.251***	.817***						
**Economic/financial CSR dimension**																			
13. Economic IS	3.304	.851	.052	.110*	.196***	.163***	.093*	.057	.067	.035	-.056	.064	.112*	.096*					
14. Economic SHOULD	2.937	1.001	.220***	.144**	.191***	.172***	.059	.034	.056	.149**	.048	.163***	.346***	.334***	.335***				
**Environmental/natural CSR dimension**																			
15. Environmental IS	3.200	.838	.058	.105*	.073	.119**	.103*	.151**	.161***	.245***	.136**	.167***	.497***	.472***	.103*	.256***			
16. Environmental SHOULD	3.880	.790	-.068	.074	.131**	.230***	.291***	.366***	.306***	.171***	.098*	.189***	.138**	.170***	.128**	.132**	.262***		
**Social CSR dimension**																			
17. Social IS	3.575	.791	.137**	.181***	.160***	.099*	.142**	.100*	.144**	.192***	.062	.183***	.525***	.510***	.082	.215***	.596***	.146**	
18. Social SHOULD	4.207	.667	-.028	.163***	.215***	.172***	.279***	.219***	.313***	.158***	.032	.230***	.179***	.205***	.100*	.081	.189***	.493***	.240***

^a^ N = 524;

* p < .05;

** p < .01;

*** p < .001.

Regarding multicollinearity, our tolerance values are higher than .10 (the lowest is .314), and VIF values are way below 10 [84]. This indicates that multicollinearity is not an issue in this research.

### Research design and analysis

This study was conducted in three stages. Initially, descriptive statistics and zero-ordered correlations among variables of interest were calculated for the aggregated sample of business students from Slovenia, Croatia, and Bosnia, and Herzegovina (N = 524). In the second stage, we used the paired samples t-test to examine the gap between CSR’s current and desired state for the analyzed business schools, according to the economic, environmental, and social dimensions. Finally, we examined the impact of personal values and business school socialization on the existing and desired state of three critical dimensions of CSR. This has been done in two steps using the hierarchical regression analysis. In the first step, we included ten dimensions of personal values (Model 1). In the second step, we entered two critical dimensions of business school socialization (Model 2). Statistical calculations were performed in IBM SPSS 24.0.

## Results

The means, standard deviations, and correlations among the studied variables for the aggregated sample are presented in Table 5.

The results from paired samples t-test regarding existing and desired states of CSR are outlined in Table 6. It provides an overview of paired samples t-test results related to CSR’s existing and preferred forms. It indicates that students’ estimated current business schools’ economic orientation is excessive, while environmental and social CSR dimensions are considered too low. This provides adequate support to Hypothesis 1.

**Table 6:** Existing and desired state of CSR dimensions of business schools^a^.

	Existing state	Desired state	GAP^b^	T
Economic/financial	3.30	2.94	-.36	-7.87***
Environmental/natural	3.20	3.89	.78	15.55***
Social	3.57	4.21	.64	15.96***

^a^ N = 524;

* p < .05;

** p < .01;

*** p < .001; paired samples t-test was used.

^b^ Gap is the difference between the desired and existing state.

Table 7 outlines effects, including only business students’ values (Model 1) and personal values and business school socialization (Model 2), on existing and desired states of business schools’ CSR.

**Table 7:** Hierarchical regression results^a^.

	Economic/financial IS		Economic/financial SHOULD		Environmental/natural IS		Environmental/natural SHOULD		Social IS		Social SHOULD	
	Model 1 (Values only)	Model 2 (Values + socialization)	Model 1 (Values only)	Model 2 (Values + socialization)	Model 1 (Values only)	Model 2 (Values + socialization)	Model 1 (Values only)	Model 2 (Values + socialization)	Model 1 (Values only)	Model 2 (Values + socialization)	Model 1 (Values only)	Model 2 (Values + socialization)
**Block 1: Personal values**												
Power	-.045	-.046	.153**	.126*	-.018	-.062	-.048	-.058	.055	.006	-.118*	-.128*
Achievement	.015	.012	.008	.020	.001	.013	-.222**	-.214**	.054	.082	.050	.060
Hedonism	.138*	.133*	.097	.047	-.017	-.098*	.011	-.004	.065	-.032	.070	.054
Stimulation	.102	.102	.104	.104	.071	.068	.126*	.126*	-.027	-.025	-.016	-.017
Self-direction	.029	.028	-.058	-.059	-.025	-.024	.168*	.169*	.058	.057	.151*	.153*
Universalism	-.034	-.033	-.069	-.079	.006	-.012	.190**	.184**	-.072	-.098	-.057	-.063
Benevolence	.033	.036	.014	-.002	.000	-.021	.171**	.161*	.078	.052	.304***	.294***
Conformity	.052	.047	.128	.091	.214**	.147*	-.015	-.027	.130	.059	-.090	-.100
Tradition	-.118	-.117	-.054	-.031	-.020	.021	-.045	-.036	-.092	-.053	-.160**	-.152**
Security	.003	.000	.039	.007	.054	-.006	.057	.046	.068	.008	.190**	.179**
**Block 2: Socialization in business schools**												
Development of knowledge		.067		.146		.318***		-.005		.284***		-.012
Culture development		-.033		.157*		.224**		.104		.281***		.108
N	524	524	524	524	524	524	524	524	524	524	524	524
R^2^	.054	.056	.090	.164	.060	.295	.190	.199	.072	.326	.176	.184
Model F	2.555**	2.197*	4.450***	7.267***	2.769**	15.200***	10.304***	9.049***	3.395***	17.470***	9.446***	8.303***

^a^ Standardized regression coefficients are shown.

* p < .05;

** p < .01;

*** p < .00.

Hypothesis H1.1 examines relations of students to the economic dimension of business schools’ CSR, with the results of hierarchical regression (Table 7) showing that it can be predicted based on students’ hedonism value only. On the other hand, the ideal state of economic CSR dimension (see column ‘economic/financial SHOULD’) replaces hedonism with the value of power and the business school socialization, related to the development of prosocial attitudes. This finding shows that the new student generations associate the current economic orientation of the business schools with hedonism, which could be projected to the perceived goals of business education (i.e., achieving economic prosperity and enjoying life). This needs to be further analyzed by additional studies, but the other finding of ‘what should be’ indicates that business students are not power-hungry, leading to their emphasis on the CSR economic dimension, but rather that they wish to balance the achieving of power with the prosocial outcomes of their education. This is an encouraging empirical finding, supporting Hypothesis 1.1. and hinting that new student generations want to use the economic dimension of CSR to obtain power for implementing change and achieving prosocial objectives.

The previous results also support Hypothesis 1.2. Namely, while conformity and the business school socialization effects are driving the current students’ perception of the environmental/natural CSR orientation (‘Environmental/natural IS’), the perception of ‘what should be’ (i.e. ‘Environmental/natural SHOULD’) shows that students wish to avoid all aspects of socialization. According to the students’ perceptions, the ideal environmental CSR would be drawn from their values only, with the logical negative influence of achievement and the predominant positive impact of universalism and benevolence.

However, when it comes to the existing social dimension of CSR, only the business school socialization influences seem to be its drivers, as business students do not seem to associate any personal values with social issues. This could be an interesting finding, as it might imply that generational characteristics, such as orientation toward technology and social networks, have shifted the attention of new student generations to feeling strongly about environmental issues and neglecting the social ones. Literature on the recent youth social movement, sometimes described in terms of social dissent [85], could support the need for further research related to this finding.

Although the influence of business schools, in terms of their socialization outcomes, seems to be a legitimate way to drive the students’ perceptions of social issues, their administrators should be aware that too much institutional pressure on ‘what should be’ will not do the job. As in the case of the environmental dimension of CSR, the ideal social dimension of CSR (‘Social SHOULD’) is driven by personal values only, while socialization influences are entirely left out of the picture. It seems pretty logical that the social dimension of CSR should be driven by benevolence and security. However, it is interesting to note that tradition is perceived as a negative factor when all three countries are considered a representative ‘mix’ of the entire region. Due to the previously discussed contextual factors, this finding could vary across the CEE and SEE region(s). Although the national comparison of CSR and the related factors across the region(s) are not within the scope of this study, such a course of analysis would be interesting in further research.

In both CSR’s environmental and social dimensions, the business school students seem to emphasize their values and wish for fewer socialization influences. This supports our Hypothesis H1.2, related to the perceived role of business schools in influencing students’ CSR attitudes, which should be seeking authenticity, based on students’ values and concerns, instead of using socialization to project the prosocial values, which the students could consider as imposed.

## Discussion

The main objective of this paper is to examine the contextualized interpretation of CSR, as related to new generations of business school students, with some generalizable findings to be applied across a range of other HEIs. The fundamental result of this study is the empirically confirmed students’ mental gap between the perceived current and expected CSR states, which should be framed within the personal and social context(s). On the one hand, in developed ‘Western’ societies, the normative definition of the CSR goals has been widely accepted by using education and the socialization processes [26]. It can be reasonably expected that CSR would be acceptable to the social majority, which assumed that some of their interests would be subjugated to the broader social goals of modern society. As new generation cohorts show reflection and doubt about the proclaimed social norms in general [86], this impedes their active participation in organizational CSR activities while showing strong ethical preferences on the personal level [39]. Our results could lead to an interpretation of these empirical results, based on the developed individual consciousness of environmental and social CSR, which has not been fully integrated into a holistic view of CSR, either on the level of organizations, relevant for young people or in the society as a whole.

The increasing individualistic orientation of young people also emphasizes the differences between personal and organizational/social CSR goals [21], which creates a social landscape, supporting the growth of the contextualized view of CSR [87]. This calls for a more adjusted, bottom-up approach to CSR development [26], which is needed to avoid the generational cynicism evidenced by a sizeable mental gap in students’ perception of their business schools’ CSR.

Different reasons could be attributed to such a result. However, they may vary considerably across the CEE and SEE region(s) due to various socio-economic circumstances, previously presented in Table 1. On the one hand, in Slovenia, which could be compared to the ‘Western’ countries, young student populations might not have faced the radical worsening of economic circumstances in their lifetime and living in an environment characterized by growing prosperity [88], which affects the interpretation and importance of the economic success. Financial support from families and occasional work opportunities might enable students to achieve relatively stable and satisfactory living standards, lowering their general interest in economic success [53]. In addition, the developed market economy, offering affordable products, might form an illusion that the availability of products for everyday life will not ever become questionable [67]. This is evidenced by the value research, which shows that values, directly or indirectly connected and dependent on financial success, such as wealth, personal success, social recognition, and exciting life, are reported as empirically significant [39].

On the other hand, the majority of the SEE region, still not accepted into the mainstream of the European integration, represented by Bosnia & Herzegovina in our sample, is characterized by a different outlook on life. As shown by Table 1, populations of those ‘undeveloped’ countries and societies feel happier, more fulfilled, and satisfied personally. This is in line with the previous findings of social research on SEE communities, societies, and social capital [28]. While lacking public trust, these populations seem to accept the need for public institutions. They have strong sentiments for economic growth and a more emphasized role of government in social and economic life. These context characteristics could be explained by a general distrust of the ‘new’ institutions, developed after the socio-economic transition of the 1990s and 2000s. If perceiving the existing social order as unjust and hypocritical, the business schools and the entire HE system could represent a significant component of the elite, generally disrespected by the new generations. The disrespect and disillusionment, followed by wishing for a different socio-economic order, might be the reason for those populations to also wish for a higher level of government ownership and intervention. In such social circumstances, aspirations toward personal and social fulfillment could be perceived as difficult or impossible to obtain, leading to a high level of young graduates’ migrations to more economically developed EU countries and societies [89].

Croatia could be more of a case of an ‘unfinished transition’, in some aspects more similar to the SEE countries, still waiting for the EU accession [90, 91], than to the representative countries of Central East Europe, becoming member states in the 2004 wave of the EU enlargement. Based on cultural and socio-economic context (see Table 1), its population could fit the description of failed convergence expectations, leading to framing their life experiences in terms of victimization [92]. Although such a proposition needs to be verified by future research, it could explain the most pessimistic outlook toward life and the lowest confidence in public institutions among the three countries included in our sample. These contextual circumstances might lead to the rejection of the CSR narratives provided by business schools and other HEIs, which might be perceived as constituencies of the ‘corrupt elite’, similar to the case of B&H students represented in our sample.

It seems that the mental gap construct is useful for empirical research of CSR perceptions, regardless of the socio-economic circumstances, and that it can be used to contextualize the empirical results in different environments. While comparative research has not been the aim of this study, the mental gap could prove attractive to the researchers, comparing and explaining CSR-related attitudes, expectations, and behaviors in different organizational and social contexts. However, it should be noted that the proposed interpretation of the empirical findings presented in this discussion is hypothetical and loosely based on extant literature. Those are to be used for the initial assessment of the mental gap research construct. Future research needs to verify the exact implementation of this construct in different national and regional contexts.

Students evaluated environmental and social dimensions of CSR higher than the economic ones. In previous studies, this proved to be more of a personal orientation and less perceived as an essential social value [26, 88], as those seem to be based on personal values and concerns [20, 87]. Our empirical research reveals that personal values should be driving CSR’s environmental and social dimensions instead of conformism and strong socialization influences, implying that students are looking for more personal freedom to create their CSR views and appreciate experiential teaching and learning models.

These findings can also be discussed from varying viewpoints of national and regional contexts. On the one hand, in the ‘Western’ countries, the obtained empirical results could be explained by the increasing individualization and relativization of the traditional CSR attitudes and behaviors. On the other hand, post-transitional social contexts of CEE and SEE countries might provide a different route to explaining the personal values as drivers of CSR’s environmental and social dimension due to the high levels of cynicism and rejection of the existing ones socio-economic order.

### Implications for theory and research

This paper has several theoretical implications. Firstly, the study focuses on the previously unused concept of a mental gap between perceived and desired CSR levels (labeled as ‘IS’ and ‘SHOULD’) in higher education. Prior studies reported states and effects of values [38–41], institutional factors [21], and situational circumstances [93] on students’ CSR. However, they were grounded on a presumption that higher education socializes students, according to prevailing CSR presumptions in broader society, using pedagogical processes [85]. This study opens a new perspective of analyzing students’ values and attitudes by recognizing the students’ mental gap of the CSR, which originates from a contrast between personal values and the socialization outcomes.

In addition, this paper conceptualizes the CSR concept among students of new student cohorts, which will be leading the development of organizational’ CSR in the following decades. It follows the existing research of value systems [35, 94] and recognizes them as a dominant factor in forming the students’ perceptions of ‘what should be’. Namely, based on the obtained empirical results, it is pretty clear that such socialization influence will decrease as the current students are empowered to interpret and influence the CSR processes in organizations and broader society. Our discussion of empirical results also shows that the mental gap construct also seems to be useful for their contextualization in various socio-economic circumstances. Therefore, it might be of interest for future research of contextualized CSR in higher education,

### Implications for academic practice

The empirical arguments in favor of the contextualized CSR view imply that higher education stakeholders need to change the context and forms of CSR education by using different CSR intervention approaches [68–70] to address subjectivization and individualization of ethics and CSR [95]. Business schools and other higher education institutions should also acknowledge a unique stream of students’ values, including personal freedom and achieving success [21, 25]. This implies that students’ pro-environmental and prosocial behavior, currently identified on the individual level [87], could be converging to create new patterns of a holistic CSR at the organizational and social level(s).

To achieve this goal, business schools need the right CSR policies and practices. Our empirical results indicate that business schools, striving to balance their CSR with the commitment to producing value, in exchange for public funds received, might not be choosing the suitable CSR targets and activities or that students misinterpret those. Such an interpretation follows advice previously provided to academic administrators by Benneworth, Pinheiro, and Sánchez-Barrioluengo [96], on understanding the social context of their institutions’ CSR, instead of adopting uninformed practices from the ‘world class’ institutions and systems. More emphasis should be placed on the students’ feelings of authenticity and their more active involvement in creating/supporting CSR-related attitudes and behaviors. Those should not be imposed by using the socialization process, but instead discovered and negotiated, within the limitations of students’ lifestyle(s) and social context(s). It is also essential to avoid the interpretation of business schools as cynical and self-absorbed, up to the level of being interpreted as hedonistic, as students seem to wish for a balance between achieving power and prosocial educational outcomes.

The disparity of attitudes to environmental and social dimensions of CSR, which seems to be driven by the global youth activism on climate change, popularized over social networks and other means of electronic communications [85], has to be considered. Business schools and other HEIs could provide an additional perspective toward understanding and meeting the needs of socially deprived people in the local communities, who might not have the capacity to voice them by using social networks and other Internet technologies.

### Limitations and future research

Our research has several limitations. Firstly, the generalization of findings may be limited due to the samples of students from countries with similar historical and social contexts [97]. However, we recognize that by avoiding comparative analysis of differences between students’ samples from each county. Nevertheless, we tried to develop a framework for discussing the obtained results in different socio-economic and cultural contexts of the three countries involved in our empirical research. We believe those could be useful in contextualizing the results and guiding future comparative analysis involving the mental gap construct.

Similar social development of other (post)transitional countries in Europe [73], broaden the area for generalizing our results to other Central European and Baltic countries. When considering the similarities of generational cohorts across countries, the generalization opportunities could be even higher, which should be done in future research.

Accepting our proposition of contextualized CSR view in business schools, which could be generalizable across different HEIs, also requires a shift of future research and practical approaches to CSR in other organizations, accepting members of new generational cohorts. As this study addressed only a part of the spectrum of influences to students’ CSR views, future research needs to check the impact of personal characteristics, previously established as relevant [87], to this newly established paradigm.

These limitations call for replication of our empirical research in various organizational and social contexts to ensure that the pattern of empirical results is valid beyond the empirical context of business schools in Central and Southern Europe. It would also be beneficial to analyze further students’ CSR concerning the changing social circumstances and other social context variables and compare the CSR orientation among different generational cohorts.

## Supporting information

S1 Dataset Supporting data file. (XLSX)
