# Correction: Students’ values, professional socialization and the mental gap of corporate social responsibility perceptions

**DOI:** 10.1371/journal.pone.0294197

**Published:** 2023-11-03

**Authors:** Nikša Alfirević, Vojko Potočan, Zlatko Nedelko

There are errors in the Funding section. The correct Funding statement is: This research has been supported by the bilateral science projects grants of the Ministry of Education, Science and Sport of the Republic of Slovenia, Slovenian Research Agency, and Ministry of Science and Education of the Republic of Croatia [no grant number assigned; project entitled How do Slovenian and Croatian higher education organizations follow goals of the European higher educational systems, concerning social responsibility?]. Authors acknowledge additional financial support from the project “Entrepreneurship for Innovative Society” (P5-0023) financially supported by the Slovenian Research Agency and Croatian Scientific Center of Excellence for School Effectiveness and Management.The funders had no role in study design, data collection and analysis, decision to publish, or preparation of the manuscript.
